# A Noninvasive Method to Determine the Fate of Fe_3_O_4_ Nanoparticles following Intravenous Injection Using Scanning SQUID Biosusceptometry

**DOI:** 10.1371/journal.pone.0048510

**Published:** 2012-11-12

**Authors:** Wei-Kung Tseng, Jen-Jie Chieh, Yi-Fan Yang, Chih-Kang Chiang, Yuh-Lien Chen, Shieh Yueh Yang, Herng-Er Horng, Hong-Chang Yang, Chau-Chung Wu

**Affiliations:** 1 Institute of Electro-optical Science and Technology, National Taiwan Normal University, Taipei, Taiwan; 2 Division of Cardiology, Department of Internal Medicine, E-Da Hospital, Kaohsiung, Taiwan; 3 Department of Medical Imaging and Radiological Sciences, I-Shou University, Kaohsiung, Taiwan; 4 Department of Internal Medicine, National Taiwan University Hospital, Taipei, Taiwan; 5 Department of Anatomy and Cell Biology, College of Medicine, National Taiwan University, Taipei, Taiwan; 6 Department of Electro-optical Engineering, Kun Shan University, Tainan, Taiwan; 7 Department of Primary Care Medicine, College of Medicine, National Taiwan University, Taipei, Taiwan; Institute of Clinical Medicine, National Cheng Kung University, Taiwan

## Abstract

Magnetic nanoparticles (MNPs) of Fe_3_O_4_ have been widely applied in many medical fields, but few studies have clearly shown the outcome of particles following intravenous injection. We performed a magnetic examination using scanning SQUID biosusceptometry (SSB). Based on the results of SSB analysis and those of established *in vitro* nonmagnetic bioassays, this study proposes a model of MNP metabolism consisting of an acute metabolic phase with an 8 h duration that is followed by a chronic metabolic phase that continues for 28 d following MNP injection. The major features included the delivery of the MNPs to the heart and other organs, the biodegradation of the MNPs in organs rich with macrophages, the excretion of iron metabolites in the urine, and the recovery of the iron load from the liver and the spleen. Increases in serum iron levels following MNP injection were accompanied by increases in the level of transferrin in the serum and the number of circulating red blood cells. Correlations between the *in vivo* and *in vitro* test results indicate the feasibility of using SSB examination for the measurement of MNP concentrations, implying future clinical applications of SSB for monitoring the hematological effects of MNP injection.

## Introduction

Nanotechnology has recently experienced significant breakthroughs in medical applications, such as contrast media for magnetic resonance imaging (MRI) [1], [2], immunoassays [3]–[5], hyperthermia [6]–[7], and drug delivery [8]–[9]. With or without bioprobe coatings for specific targeting, magnetic nanoparticles (MNPs) composed of Fe_3_O_4_ are promising nanocarriers that demonstrate nontoxicity and superior magnetic characteristics when using well-established methodologies [2], [4]. By contrast, although nontoxic, Gadolinium-containing nanoparticles have induced clinical problems, such as renal failure [10]. Therefore, a metabolic model is required for evaluating iron oxide MNPs. Intravenously injected MNPs remain in the bloodstream for a limited duration before being degraded or excreted [11]. Thus, metabolic studies of MNPs should include evaluations of pharmacokinetics [12], biodegradation [13], and excretion [14] to determine how these factors may influence the therapeutic effects, side effects, and potential adverse reactions resulting from injected MNPs. Moreover, the dynamic metabolic condition of individual patient should be monitored noninvasively to ensure the safety of MNP injections.

Although the metabolism of MNPs has been investigated [12]–[17], the number of methodologies available for MNP -related examinations has been limited, and conclusive findings are minimal. The methods used in previous studies can be classified as either magnetic [13], [16], [17] or nonmagnetic examinations [12], [14], [15]. Nonmagnetic examinations include tests for serum iron concentrations, hematocrit (HCT) tests, and determining the distribution of iron content in organs using Prussian blue staining. Different iron ions and iron-containing compounds have distinct magnetic characteristics. Thus, magnetic examinations involve identifying iron metabolites based on variations in magnetic properties. Except for magnetic resonance imaging (MRI), alternating current (AC) magnetic susceptometry [17]–[18] is currently the most sensitive method available for measuring variations in magnetism when preformed using a superconducting quantum interference device (SQUID) magnetometer. Both MRI and AC magnetic susceptometry have limitations. Although MRI can measure magnetic particles *in vivo*, it is a costly procedure and the availability of MRI instruments is limited. By contrast, AC magnetic susceptometry is limited to *in vitro* testing because it requires drying a sample and examining it in a vacuum at low temperature.

Recently, a novel scanning SQUID biosusceptometry (SSB) method was developed to track MNPs at room temperature both *in vivo* and *in vitro*, based on AC susceptibility [19]–[20]. The method has been demonstrated high specificity for MNPs with superparamagnetic properties. However, whether other biodegraded forms of iron existed in these previous studies remains unclear because the majority of the biodegraded forms, such as iron ions or ferritins in liver tissue, have extremely low paramagnetic or diamagnetic properties rather than superparamagnetic ones [19]–[20].

The objective of this study was to establish a comprehensive model of MNP metabolism by conducting both *in vivo* and *in vitro* tests, including magnetic examination by using SSB and nonmagnetic examinations that are based on well-established procedures. We also evaluated the feasibility of the magnetic tests involving SSB as an *in vivo* examination to monitor the dynamic effects associated with MNPs, thereby providing further evidence to support future clinical applications of these methods.

## Materials and Methods

All of the experimental procedures were approved by the Animal Care and Use Committee of National Taiwan University College of Medicine (No. 20110009).

### Characterizing MNPs

The MF used in this study consisted of MNPs of dextran-coated Fe_3_O_4_ (MF-DEX-0060; MagQu, New Taipei City, Taiwan, ROC). The MNPs were purified using a co-precipitation procedure [21], in which the average size and size distribution of the Fe_3_O_4_ magnetite nanoparticles were controlled using ammonium hydroxide and urea to manipulate the pH of the ferrite solution homogeneously. Dextran was selected as a surfactant for the nanoparticles because of its nontoxicity, hydrophilicity, and high affinity for biological macromolecules.

The size distributions of the MNPs were measured using a Nanotrac particle size analyzer (Microtrac, Montgomeryville, PA, USA). The magnetism saturations of the MNPs were measured using a vibrating sample magnetometer (EG&G PARC, Newnan, GA, USA). The stability test was performed by observing the size distributions of the MNPs for more than 1 y.

### Animal Methods

Five-week-old male Wistar rats (Laboratory Animal Center, National Taiwan University, Taipei City, Taiwan, ROC) with a total body weight of 250–300 g were administered through the tail vein a single 0.3-ml intravenous injection of MF (0.9 emu/g) that was equivalent to 7.1 mg MNPs/kg body weight, as previously described [12], [15], [16]. Table 1 lists the time points at which the different *in vivo* and *in vitro* examinations were performed.

**Table 1:** Experimental Approach.

Examination Type	Specimen	Methodology		Rat number/Time points for scanning examination
***in vivo*** ** test**	**Heart**	• Magnetic measurement by SSB		2 rats per each time point of 0 h, 0.5
				h, 1 h, 2 h, 4 h, 8 h, 1 d, 3 d, 5 d, 1
				wk, 2 wk, and 4 wk
	**Liver**	• Magnetic measurement by SSB		2 rats per each time point of 0 h, 1 h,
				2 h, 4 h, and 8 h
***in vitro*** ** test**	**Blood**	• Magnetic measurement by SSB		5 rats per each time point of 0 h, 0.5
		• Serum iron test		h, 1 h, 2 h, 4 h, 8 h, 1 d, 3 d, 5 d, 1
		• HCT test		wk, 2 wk, and 4 wk
		• TIBC test		
		• Transferrin test		
	**Liver**	• Prussian blue staining		5 rats per each time point of 0 h, 0.5
		• Magnetic measurement by SSB		h, 1 h, 2 h, 4 h, 8 h, 1 d, 3 d, 5 d, 1
		• ICP test		wk, 2 wk, and 4 wk
		• TEM test		5 rats per each time point of 0 h, 2 h,
				8 h, 1 d, and 4 wk
	**Spleen**	• Prussian blue staining		5 rats per each time point of 0 h, 0.5
		• Magnetic measurement by SSB		h, 1 h, 2 h, 4 h, 8 h, 1 d, 3 d, 5 d, 1
		• ICP test		wk, 2 wk, and 4 wk
	**Lung**	• Prussian blue staining		5 rats per each time point of 0 h, 0.5
		• Magnetic measurement by SSB		h, 1 h, 2 h, 4 h, 8 h, 1 d, 3 d, 5 d, 1
		• ICP test		wk, 2 wk, and 4 wk
	**Kidney**	• Prussian blue staining		5 rats per each time point of 0 h, 0.5
		• Magnetic measurement by SSB		h, 1 h, 2 h, 4 h, 8 h, 1 d, 3 d, 5 d, 1
		• ICP test		wk, 2 wk, and 4 wk
		• TEM test		5 rats per each time point of 0 h, 2 h,
				8 h, 1 d, and 4 wk

For the *in vitro* analyses, 5 rats were euthanized at each time point following MF injection. The 5 rats representing the control group were euthanized at 0 h and did not receive an MF injection. The liver, spleen, lung, kidney, and blood specimens were examined during *in vitro* analysis by using both magnetic and nonmagnetic methods. The magnetic *in vitro* measurements were performed using SSB. The nonmagnetic *in vitro* examinations included Prussian blue tissue staining, the inductively coupled plasma (ICP) test, serum iron test, total iron-binding capacity (TIBC) test, transferrin test, HCT test, and transmission-electron microscopy (TEM) imaging. TEM analysis was limited to samples collected at 0 h, 2 h, 8 h, 1 d, and 4 wk.

For the *in vivo* analyses, 2 rats were anesthetized with an intraperitoneal injection of pentobarbital (30 mg/kg) at each time point following the MF injection (Table 1). Two anesthetized rats representing the control group were analyzed prior to receiving the MF injection (0 h). The magnetic measurements of the heart and liver were performed using SSB across the chest and abdomen, respectively. The feasibility of using SSB for MNP diagnostics was validated by comparing the results of the *in vivo* and *in vitro* tests.

### Magnetic Measurements

**Figure 1:**
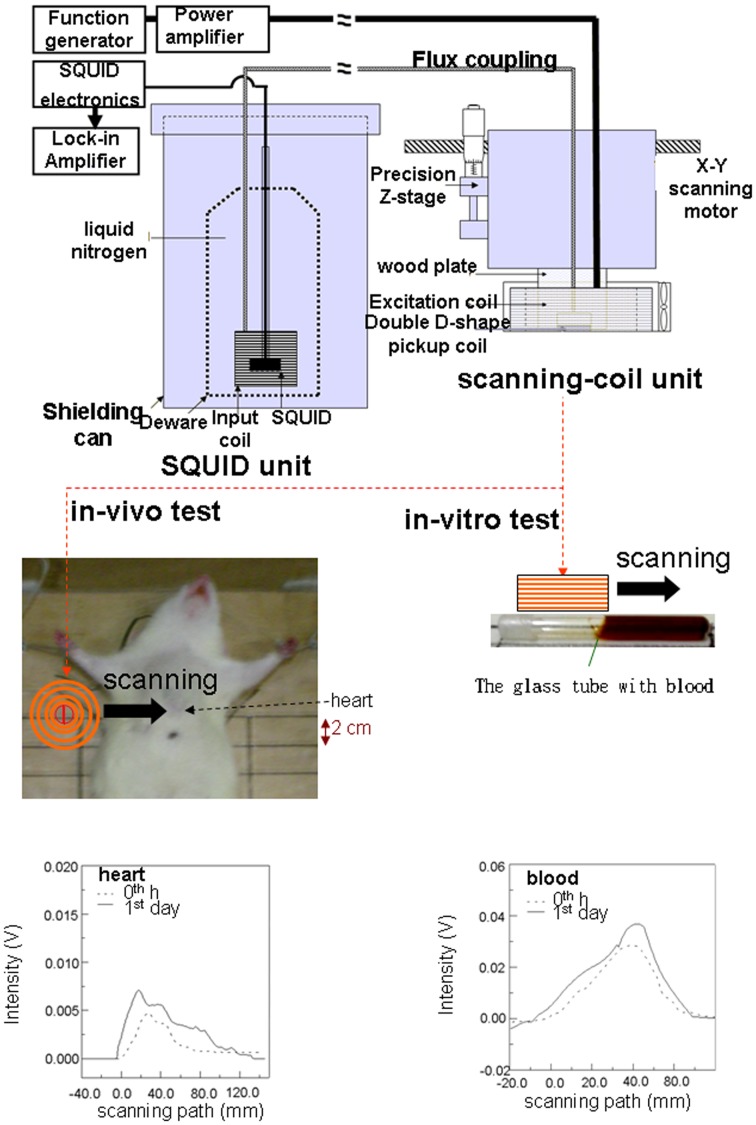
*In vivo* and *in vitro* examinations by magnetic SSB. Chest scans were performed for the *in vivo* examination on the heart (left lower panel), and tubes containing blood (right lower panel) drawn from the hearts of euthanized rats were scanned by SSB for the *in vitro* examination.

The design of our SSB instrument was based on configurations that have demonstrated high sensitivity for AC susceptibility of MNPs in previous studies [19]–[20]. The SSB instrument was composed of a SQUID unit and a scanning-coil unit that were connected between an input coil and pickup coils, based on the concept of flux coupling (Figure 1). The SQUID unit consisted of a high Curie-temperature SQUID sensor (RF magnetometer, Juelicher SQUID GmbH, Juelicher, Germany), a Dewar flask (MVE Lab10, Cryo Solutions Corp., Hertogenbosch, the Netherlands) containing liquid nitrogen, a shielding can (MagQu Corp., New Taipei City, Taiwan) to reduce surrounding noise, and an input coil. The scanning-coil unit contained a hand probe consisting of a cylindrical excitation coil and a centered double D-shaped pickup coil, as well as a 3D positing mechanism for the X-Y scanning motor and a precision Z-stage (ONSET ELECTRO-OPTICS Corp., Taipei City, Taiwan). Continuously static measurements of the liver tissue and blood samples had previously demonstrated significant correlations with *in vivo* and *in vitro* examinations within 8 h of MF injection, and a similar agreement was also reported between *in vitro* and ICP tests of liver tissue up to 4 h following injection [19]–[20]. Based on these reports, we concluded that the results of continuously static and *in vivo*/*in vitro* SSB examinations of MNPs within 8 h of MF injection were reliable for the comparison with those obtained by intermittent dynamic examinations in this study.

The *in vivo* magnetic examinations in this work were performed by passing the scanning-coil unit across the chest and abdomen, approximately 2 cm above and below the xiphoid, respectively. This intermittently dynamic *in vivo* magnetic examination differed from the continuously static and *in vivo* measurements that we performed in preliminary studies [19]–[20]. However, the expression of both sets of *in vivo* data using ΔM were normalized to the maximum level (ΔM/M_max_) for comparison, because the MNP distribution in the rat body is too complex to simulate the known magnetism of different individuals (Figure 1).

The *in vitro* magnetic examinations in our current investigation were similar to published results of our preliminary studies [19]–[20], in which a glass tube that contained the homogenized tissue from the euthanized animal was scanned (Figure 1). Briefly, 0.3 g of tissue was homogenized using a YCK-100 homogenizer (Yeong-Shin Corp, Hsinchu City, Taiwan), and mixed with 0.4 ml of normal saline. For examining the liquid samples, 0.3 ml of urine and 0.6 ml of blood were sealed in glass tubes (60P650B, Kimble Chase Crop., NJ, USA) using Parafilm (M type, Pechiney Corp, Chicago, IL, USA) prior to analysis. The area under the scanning curves of the signal intensity was proportional to the magnetism, M (Figure 1) [19]–[20]. Glass tubes containing different concentrations of MF were used as the *in vitro* phantom for calibrating the instrument [19]–[20]. The magnetic concentration of the *in vitro* specimens is expressed as the unit of mass magnetization (emu/g). The magnetism difference between the post- and pre-MF injection, ΔM, was also used for analyzing the magnetism variation of the samples.

### Nonmagnetic Measurements

Three different sets of specimens were prepared for the nonmagnetic *in vitro* tests. The first set consisted of the tissue specimens for Prussian blue staining, TEM imaging, and ICP analysis. The second set comprised the whole blood samples for HCT analysis. The third set consisted of the serum samples for the serum iron, transferrin, and TIBC tests.

Prussian blue staining was used to observe the distribution of iron metabolites in the tissue, either as MNPs or iron metabolites. The Prussian blue dye contained 5% potassium ferrocyanide and 5% hydrochloric acid (Merck KGaA Corp., Darmstadt, Germany). Hydrochloric acid degrades MNPs into ferric ions, and potassium ferrocyanide reacts with ferric ions, forming a blue precipitate. The images were observed using a microscope under 1000× magnification. The number of pixels representing the blue spots within the images was normalized to the total number of pixels in the entire image area to represent the amount of iron present in the sample.

To investigate the possible relationships between the presence of MNPs and cellular processes, such as phagocytosis or cellular storage, TEM was used to examine the detailed distribution of nanoparticles within the cells. At a resolution that is significantly higher than light microscopy, TEM was performed on the animal tissues obtained at different time intervals after the MNP injection. TEM has been widely applied in cancer research, virology, and materials science.

The tissues were fixed in 2% paraformaldehyde and 2% glutaraldehyde in a 0.1-M cacodylate buffer for 4 h. The tissues were postfixed for 1 h with 1% osmium tetroxide, and dehydrated in a graded series of ethanol washes, before being embedded in Epon epoxy resin (Momentive Corp., TX, USA). Ultrathin sections on 150-mesh copper grids were lightly stained with lead citrate, and analyzed by using TEM with a Hitachi H7100 instrument (Tokyo, Japan) at an accelerating voltage of 100 kV. High-resolution digital images were obtained using a CCD camera (AMT 2.25, Advanced Microscopy Techniques, Danvers, MA, USA).

The ICP test is considered the gold standard for tracing ultralow concentrations of compounds in acid-solubilized tissue samples. The tissue samples (0.1 g) were dissolved in a 65% HNO_3_ solution (Merck KGaA Corp., Darmstadt, Germany), and the solubilized tissues were diluted prior to ICP examination. In ICP analysis, the sample was initially ionized using a plasma source inducted by time-varying magnetic fields, and the ions were then separated and quantified using a mass spectrometer (X Series II, Thermo Scientific Corp., IL, USA). The amounts of iron detected at the various time points were compared to that at 0 h (ΔC_Fe_ in ppm) to determine the amount of iron in the tissues resulting from the MF injection.

The HCT test entailed measuring the volume percentage of red blood cells in whole blood. HCT levels are used as a diagnostic tool for numerous conditions, including anemia and polycythemia. We hypothesized that the iron metabolites originating from injected MNPs may be incorporated into red blood cells during erythropoiesis, based on the results of previous studies [22]. Blood samples (0.1 ml) were drawn from the rat hearts into evacuated tubes (MBD 7835, BD Corp., NJ, USA) using ethylenediaminetetraacetic acid (EDTA) for examination. The HCT levels at the various time points were compared to the HCT measurements at 0 h (ΔHCT in µg/dl) to evaluate the effect of MNP injection on the level of circulating red blood cells.

The concentration of serum iron is an indicator of the amount of circulating ferric ions bound to transferrin, and the amount of ferric ions bound to transferrin is an indicator of the amount of free ferric ions available for transport to organs. The TIBC test involves measuring the capacity of blood to bind iron with transferrin. For the clinical diagnosis of blood disorders, serum iron and TIBC tests are generally performed simultaneously. Approximately 1 ml of serum prepared from a whole blood sample was used for the serum iron, transferrin, and TIBC tests at the various time points listed in Table 1 to determine the amount of ferric ions in the blood of rats injected with the MF relative to that of the control rats (ΔFe^3+^in µg/dl, ΔC_transferrins_ in mg/dl, and ΔC_TIBC_ in µg/dl). The serum iron, transferrin, and TIBC tests were performed by Union Clinical Laboratory (Taipei City, Taiwan).

## Results

### Reagent Characteristics

**Figure 2:**
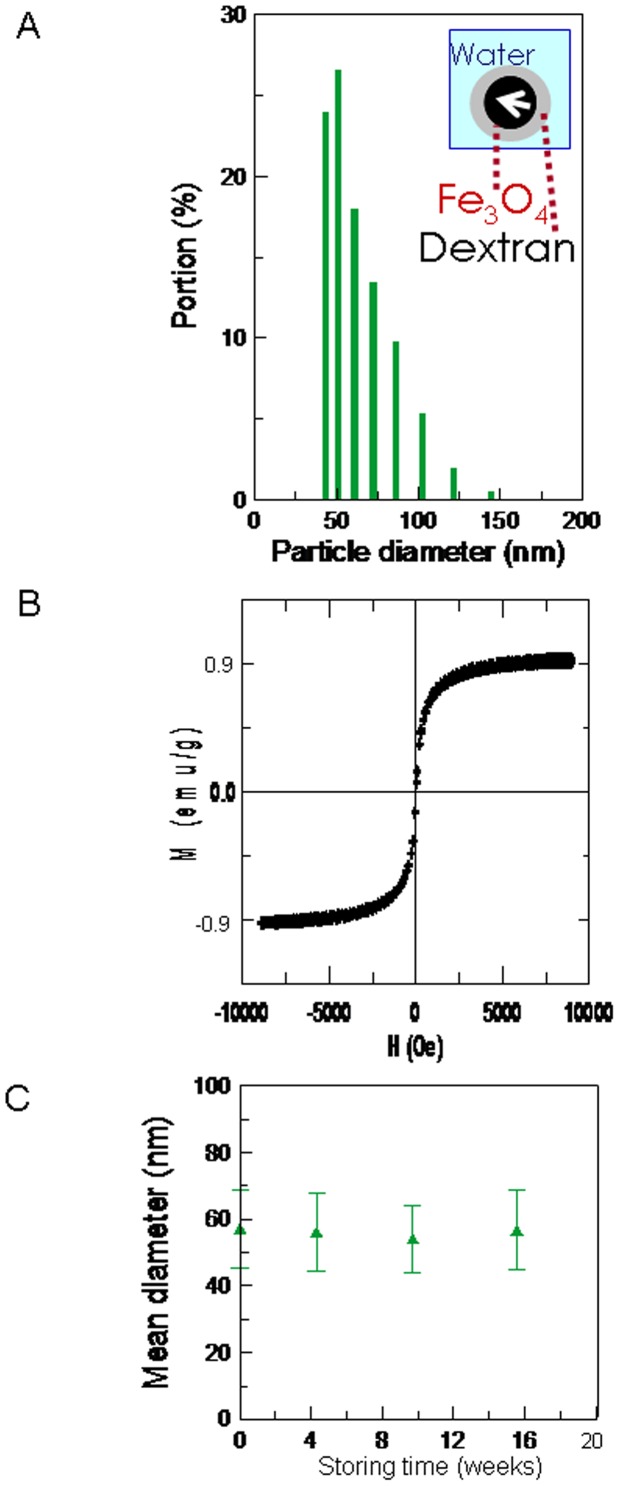
Characteristics of the MF reagent. (**A**) The particle size distribution, (**B**) the magnetism-applied field (M-H) curve of the MF reagent with a concentration of 0.9 emu/g, and (**C**) the stability of the MF reagent with a mean particle size of approximately 57 nm was monitored for 4 mo.

The mean diameter of the MNPs was 57.2±11.8 nm (Figure 2A). The M-H curve shows that the MF reagent had a superparamagnetic character (Figure 2B). The stability test showed that the MF remained stable for at least 4 mo (Figure 2C). Therefore, we concluded that the MNP degradation within 1 d following MF injection resulted from biological reactions in the animal, rather than from spontaneous degradation processes.

### *In vivo* and *in vitro* Tests

**Figure 3:**
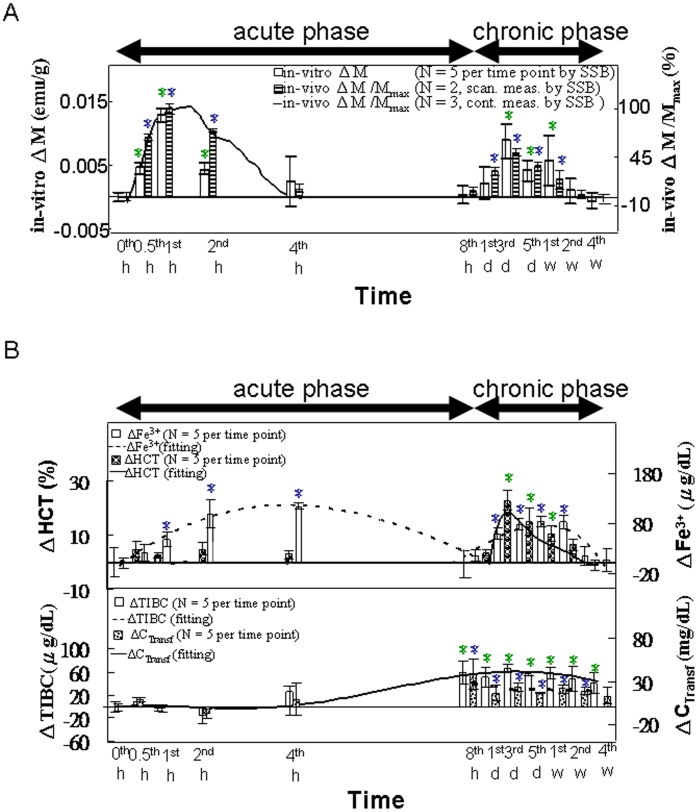
*In vivo* and *in vitro* examinations of the blood. (**A**) *In vivo* consisted of continuously scanning the heart using SSB from 0 to 4 h (n = 2) and scanning across the chest intermittently from 0 h to 4 wk (n = 2). The *in vitro* test of the blood using SSB was performed from 0 h to 4 wk (n = 5). (**B**) The *in vitro* HCT, serum iron concentration, transferrin, and TIBC tests were performed from 0 h to 4 wk (n = 5). Within the different time points in each methodology, star (*) denote significant differences in the data that are presented (*p*<0.05).

Figure 1 shows the raw data of ΔM and ΔM/M_max_, illustrated according to the scanning curves in the *in vivo* and *in vitro* tests of SSB at the 0 h and the 1 d time points. The *in vitro* magnetic examination of blood (Figure 3A) using SSB showed that the first peak of ΔM occurred within 8 h following the MNP injection, with a maximum at 1 h; this period was defined as the acute phase. The second and smaller peak occurred between the 8-h and 4-wk time points, with a maximum at 3 d; this period was defined as the chronic phase. For *in vivo* magnetic examination of the heart using SSB (Figure 3A), the variations of ΔM/M_max_ were assessed using continuous measurements in our preliminary results (solid line) [19]–[20], which occurred within 4 h post-injection, with the maximum occurring at 1 h post-injection. Similarly, the intermittent measurements (bar chart) also showed 2 separate peaks occurring during the acute and chronic phases.

Figure 3B shows that the serum iron variation (ΔFe^3+^) indicated 2 separate peaks in the acute and chronic phases, whereas ΔHCT indicated only one peak during the chronic phase. The increases in ΔTIBC and ΔC_transferrins_ began in the late acute phase and continued through the chronic phase, with each reaching a maximum at 8 h post-injection.

**Figure 4:**
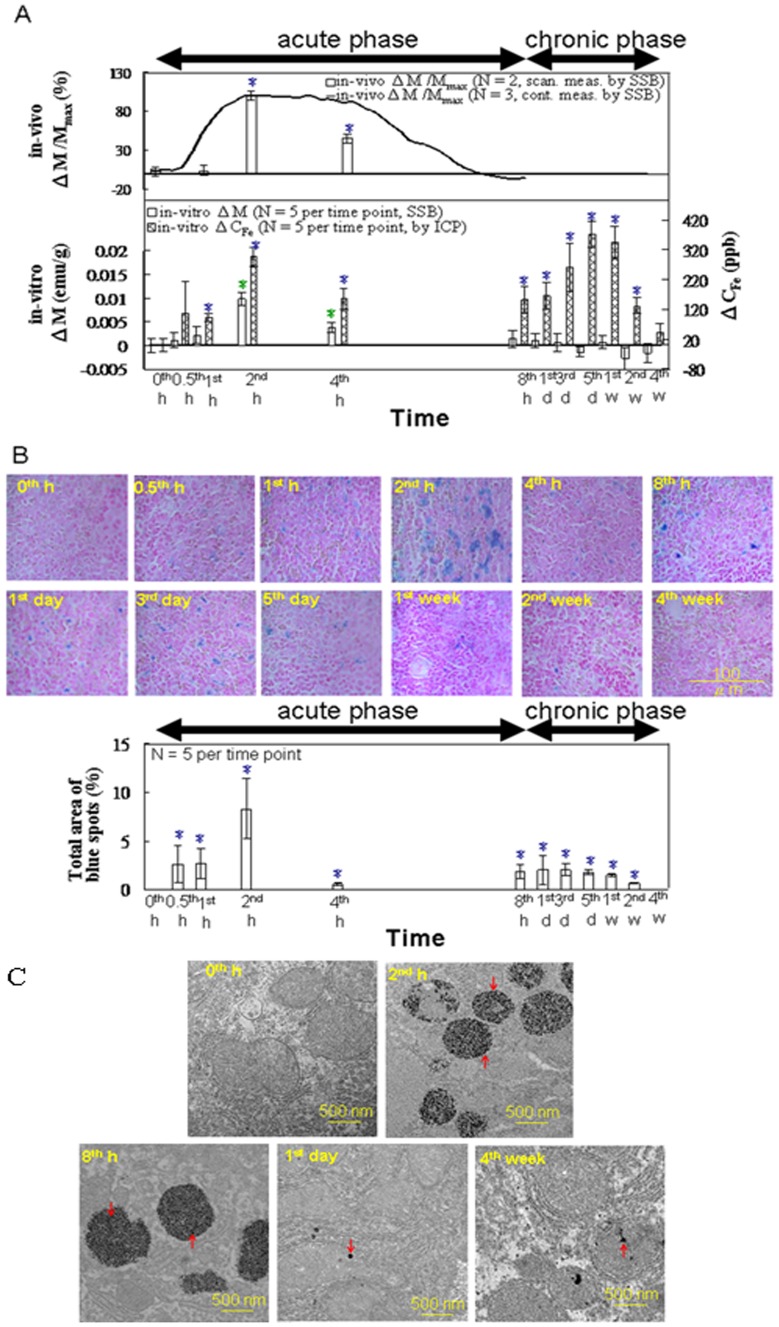
*In vitro* examination of the liver. (**A**) The *in vitro* magnetic SSB test results versus the ICP test results (from 0 h to 4 wk, n = 5). (**B**) Prussian blue staining (from 0 h to 4 wk, n = 5). (**C**) TEM images of representative macrophages in the liver tissue specimens of rats injected with the MNPs at 0 h, 2 h, 8 h, 1 d, and 4 wk. The arrows indicate the MNPs (Scale bars: 500 nm). Within the varying time points in each methodology, star (*) denote significant differences in the data that are presented (*p*<0.05).

Figure 4A shows the results of the *in vivo* magnetic examinations of the liver using SSB. The ΔM/M_max_ determined by continuous measurements in our preliminary works (solid line) [19]–[20] and by intermittent measurements in our current study (bar chart) showed variations occurring within 8 h post-injection, with a maximum at 2 h during the acute phase. For the *in vitro* tests of liver specimens by using SSB and ICP (Figure 4A), the ΔM also showed a single peak with a maximum at 2 h during the acute phase. However, the ΔC_Fe_ showed one peak during the acute phase (2 h) and another during the chronic phase (3 d, 5 d, and 1 wk). As shown in Figure 4B, the area of the Prussian blue staining reached a maximum at 2 h during the acute phase, with levels receding through 4 wk post-injection. As shown in Figure 4C, TEM analysis revealed significant increases in the number of electron-dense black particles that were present in the vacuoles of macrophages at 2 h and 8 h following MNP injection, as compared with the macrophages in the control rats. TEM analysis at subsequent time points (1 d and 4 wk) did not indicate as significant a number of particles that were in the macrophages.

**Figure 5:**
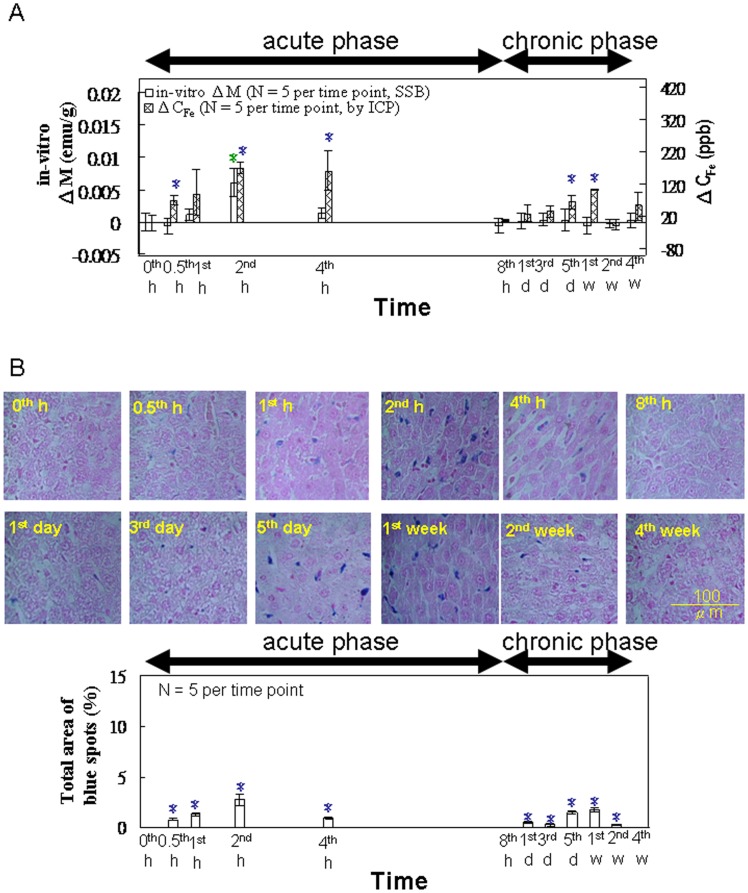
*In vitro* examination of the spleen. (**A**) The *in vitro* magnetic SSB test results versus the ICP test results (from 0 h to 4 wk, n = 5). (**B**) Prussian blue staining (from 0 h to 4 wk, n = 5). Within the varying time points in each methodology, the different letters denote significant differences in the data that are presented (*p*<0.05).

For *in vitro* tests of the spleen by using SSB and ICP (Figure 5A), both ΔM and ΔC_Fe_ showed a maximum at 2 h during the acute phase, whereas ΔC_Fe_ showed another lower maximum during the chronic phase (5 d and 1 wk). In Figure 5B, the area of the Prussian blue staining indicates one maximum at 2 h during the acute phase and another lower maximum during the chronic phase (5 d and 1 wk).

**Figure 6:**
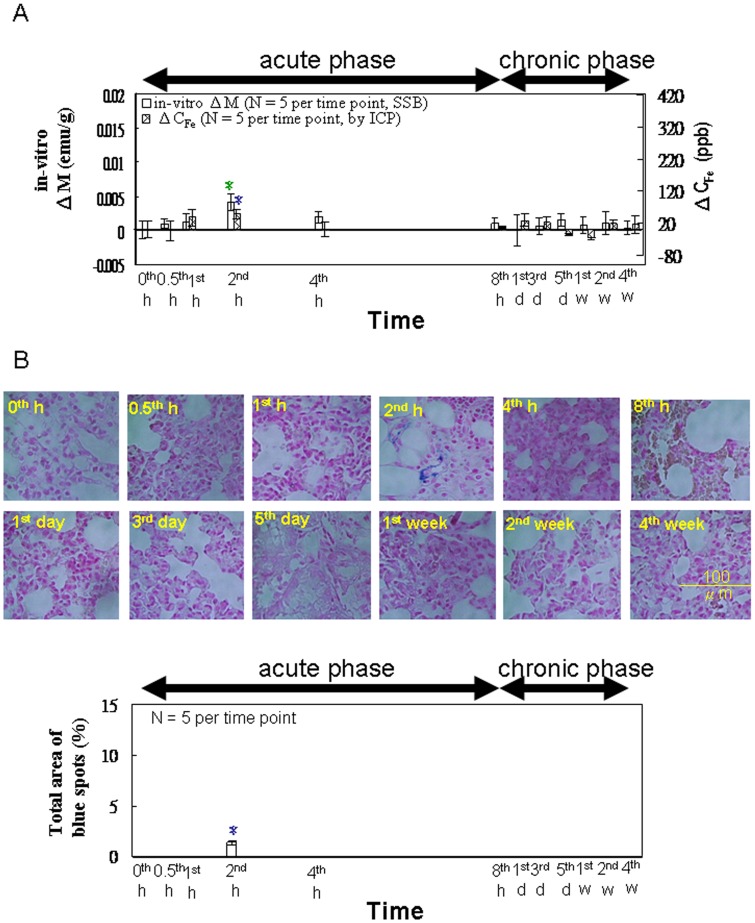
*In vitro* examination of the lung. (**A**) The *in vitro* magnetic SSB test results versus the ICP test results (from 0 h to 4 wk, n = 5). (**B**) Prussian blue staining (from 0 h to 4 wk, n = 5). Within the varying time points in each methodology, star (*) denote significant differences in the data that are presented (*p*<0.05).

For *in vitro* tests of lung specimens using SSB and ICP (Figure 6A), both ΔM and ΔC_Fe_ showed a maximum at 2 h during the acute phase, with no discernible peaks during the chronic phase. In Figure 6B, the area of the Prussian blue staining reached a maximum only at 2 h during the acute phase, and showed no discernible peaks at other time points.

**Figure 7:**
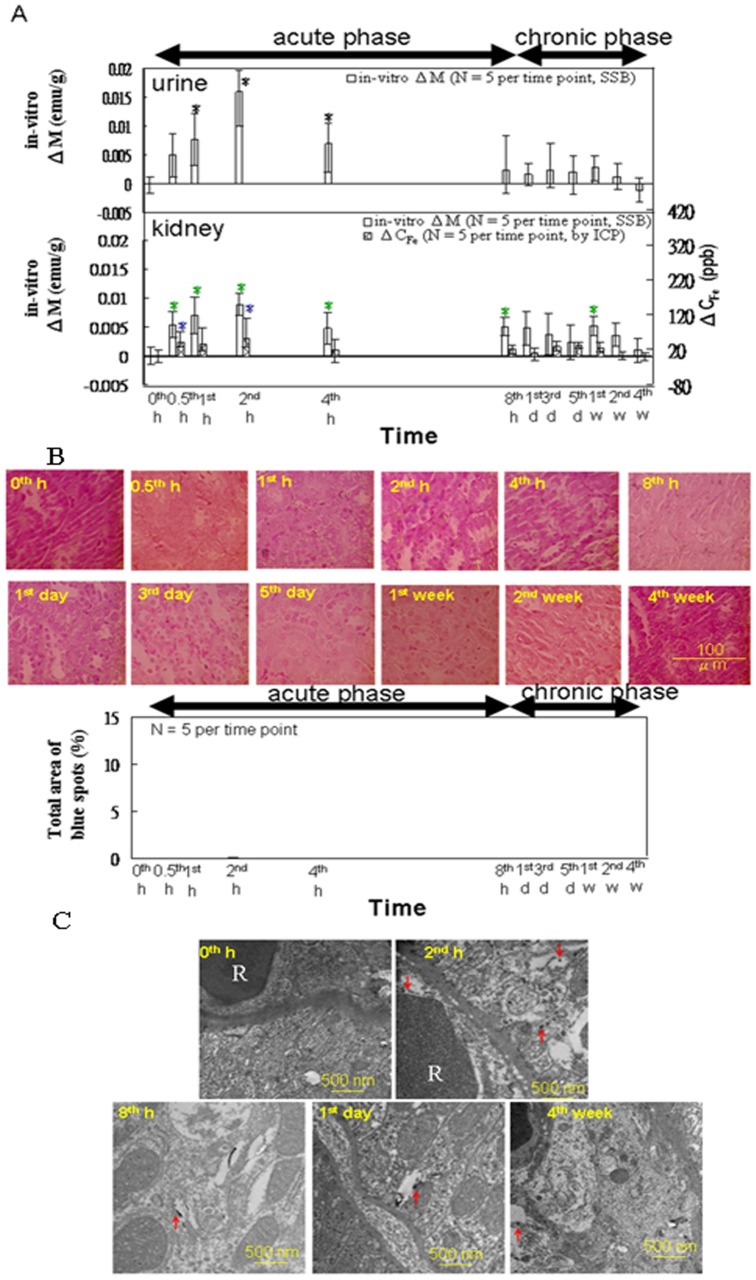
*In vitro* examination of particle excretion in the kidney. (**A**) The i*n vitro* magnetic SSB measurements of the urine and kidney tissues versus the ICP test results for the kidney tissue (from 0 h to 4 wk, n = 5). (**B**) Prussian blue staining of the kidney tissue (from 0 h to 4 wk, n = 5). (**C**) TEM images of representative macrophages in the kidney specimens of the rats injected with the MNPs at 0 h, 2 h, 8 h, 1 d, and 4 wk. The arrows indicate the MNPs (Scale bars: 500 nm). Within the varying time points in each methodology, star (*) denote significant differences in the data that are presented (*p*<0.05).

For *in vitro* tests of kidneys and urine by using SSB (Figure 7A), ΔM of both the kidney specimens and urine samples showed one peak during the acute phase. However, ΔM remained significant in the kidney specimens through the 2-wk time point during the chronic phase, whereas ΔM was not significant in the urine during the chronic phase. As shown in Figure 7B, no Prussian blue staining was observed at any time point. Figure 7C shows a significant number of large black spots in the renal sections at 2 h post-injection, as compared with sections from the control rats. A significant number of black spots were not observed in the kidney sections at 8 h and 1 d post-injection; however, some were visible in the lumen of blood vessels at 4 wk post-injection.

## Discussion

### Feasibility of the *in vivo* Magnetic Examination using SSB

As shown in Figure 3A, the double-peaked ΔM appearance of the blood results was similar to the findings of a previous study, in which magnetic Fe_3_O_4_ nanoparticles were administered similarly [12]. The *in vitro* ΔM results of blood sample analysis and the *in vivo* ΔM/M_max_ of the heart were also similar for both the continuous measurements during the acute phase and the intermittent measurements during the acute and chronic phases. These results demonstrate the feasibility of this *in vivo* magnetic examination of the heart by using SSB and the chest scan method.

### The Acute Phase of the Metabolic Model

In the acute phase, the first peak of the serum iron variation (ΔFe^3+^ in Figure 3B) occurred later than that of ΔM in the blood collected from the heart (Figure 3A). However, ΔFe^3+^ partially overlapped with that of the liver (Figure 4A), spleen (Figure 5A), and lung (Figure 6A), with the maxima for these measurements distributed at approximately 2 h and 4 h post-injection (Figures 3B, 4A, 5A, and 6A).

These results indicated that the superparamagnetic MNPs were transported through the blood within 1 h (as indicated by the peak in the blood collected from the heart) to the organs at 2 h, including the liver, spleen, and lung, as evidenced by the peaks observed in those tissues in the acute phase. Thereafter, the MNPs were biodegraded into ferrous or ferric ions. The majority of the ferrous ions were likely converted into ferric ions by ceruloplasmins in the alkaline blood [23], and then bound by transferrin, thus increasing the serum iron concentration. The released ferric irons and transferrin contributed to the first peak of serum iron concentration from 2 to 4 h in the blood collected from the hearts. During the same period, the magnetic intensity of these organs decreased because of fewer superparamagnetic MNPs in the blood and the lower magnetic AC susceptibility of the serum irons.

Similarly, the variation in ΔC_Fe_ in these organs (Figures 4A, 5A, and 6A) was the same as that in ΔM and ΔM/M_max_, indicating the MNP dynamics in these organs. In the liver (Figure 4A), the decreasing rates from 2 to 4 h were significantly different among the ΔC_Fe_, ΔM, and ΔM/M_max_ measurements. The sharper decrease in ΔM, as compared with the decrease in ΔC_Fe_, indicated fewer superparamagnetic MNPs and more iron ions with low magnetic AC susceptibility in the tissue matrix during that period. Furthermore, in the specimens from both the liver (Figure 4A) and heart (Figure 3A), the sharper decreases in the *in vitro* ΔM, as compared with those of the *in vivo* ΔM/M_max_, suggested that the MNPs were transported from the blood to the organ tissues in accordance with a 2-compartment open model.

The results of Prussian blue staining (Figures 4B, 5B, and 6B) indicated that, among the organs examined, the liver tissue showed the greatest iron density, whereas the lung tissue showed the lowest iron density. This observed trend among the organs correlates with the distribution of macrophages among these organs [24]. In addition, the intensity of staining reached a maximum at 2 h, and then decreased gradually, reflecting the ΔM variation in these organs (Figures 4A, 5A, and 6A).

In the TEM images of the liver specimens, more black nanoparticles were observed in the vacuoles of macrophages at 2 h and 8 h, as compared with the control specimens, indicating that the iron was acquired by the macrophages through the phagocytosis of the MNPs (Figure 4C). These morphological observations indicated increased macrophage activity in the hepatic tissues following the MF injection. This conclusion is also supported by the high ΔM and ΔC_Fe_ values observed for the liver specimens at 2 h correlating with the observations of many MNPs by using TEM, followed by the low ΔM and high ΔC_Fe_ values observed in the liver specimens at 8 h, presumably because of the biodegradation of the MNPs into iron ions.

These results are consistent with the findings from previous studies [15], [25]. Using an ^59^Fe radiotracer, Weissleder et al. showed that, at 1 h after administering a similar-sized superparamagnetic-iron-oxide preparation to rats, more than 80% of the ^59^Fe was sequestered in the liver, with peak concentrations of ^59^Fe occurring in the liver at 2 h and in the spleen at 4 h post-injection [22].

### The Chronic Phase of the Metabolic Model

Because most of the MNPs were degraded by the mononuclear phagocytes in tissues into ferric irons during the acute phase, it seems reasonable that the ferric irons produced by the biodegradation of the MNPs were bound by transferrin in the blood, transported to the organs, and stored as ferritins in the liver, according to the general processes for normal iron metabolism. This phenomenon could be proved by the characteristics of livers and blood.

The production of the ferric irons resulting from the biodegradation of MNPs was reflected in the peak of ΔFe^3+^ in blood samples. While ΔFe^3+^ decreased at 8 h post-injection, an increase in transferrin production is suggested by the peak in ΔC_transferrins_ and ΔTIBC those occurred from the late acute phase through the chronic phase (Figure 3B).

In general, transferrin transports iron to organs needing high amounts of iron, such as skeletal muscle and erythropoietic tissue [22]. The ΔHCT reached a peak between 3 and 7 d post-injection, and then decreased gradually to baseline levels by Week 4 (Figure 3B). The *in vitro* ΔM of the blood and *in vivo* ΔM/M_max_ of the heart (Figure 3A) varied simultaneously with the ΔHCT variation because the hemoglobin in the red blood cells contained the source of the paramagnetism. These results show that SSB is a feasible method for noninvasive, dynamic hematological evaluations of the effects of MNP injection.

Evaluating the liver specimens revealed iron storage in the liver cells based on low AC susceptibility, as indicated by the second peak of ΔC_Fe_ (Figure 4A), Prussian blue staining (Figure 4B), low ΔM (Figure 4A), and the small number of black spots in the interstitial spaces and in the cells observed in the TEM images collected during the chronic phase (Figure 4C). The black particles in the TEM images were most likely ferritin molecules with a high density of bound ferric ions, a conclusion supported by the low ΔM and high ΔC_Fe_ in the liver, as well as the high ΔFe^3+^ and high ΔC_transferrins_ observed in the blood.

Compared with observations in the liver, the analysis of the spleen tissue showed a similar lower peak from 5 to 7 d post-injection (Figure 5A, 5B). This was likely the result of the spleen’s role in the recycling of damaged and old erythrocytes (Figures 5A and 5B). This conclusion is also supported by the observation of the ΔHCT peak occurring from 3 to 7 d returning to baseline levels following that period (Figure 3B), as well as the lack of similar peaks observed for the lung specimens (Figure 6) because the lung is not a major organ for iron metabolism or storage.

These findings are consistent with those of several previous reports in that serum iron concentrations reached a second peak between 1 and 5 d, a peak in hemoglobin levels occurred during the same period, and a second peak of hepatic iron occurred from 3 to 7 d following iron injection [12], [22], [25]. Another previous study also reported that essentially no iron accumulation was observed in the liver at 4 wk after intravenous injection of a similar amount of iron in a Wistar rat model [26].

### Excretion Mode

In the acute phase, a major peak in △M was observed for both the urine and kidney specimens, and a peak was observed in △C_Fe_ for the kidney (Figure 7A). These results demonstrated that the level of circulating MNPs peaked at approximately 2 h, reaching the kidneys, from which they were excreted into the urine. The absence of Prussian blue staining was essentially observed in the kidney tissue (Figure 7B), which would not indicate the presence of iron metabolites originating from the MNPs, may have resulted from the rate of glomerular filtration occurring at a level high enough to prevent the deposition of the Prussian blue precipitant in the kidney tissue [14]. This conclusion is supported by the presence of dark particles in the lumen of glomerular blood vessels and in the cytoplasm of renal tubular cells observed in the TEM images of renal sections obtained at 2 h post-injection.

Significant variations in the △M and the △C_Fe_ measurements for the kidney between 3 and 7 d indicate that iron metabolites were also excreted from the kidney during the chronic phase [27]. These observations are consistent with the release of iron metabolites into the blood from iron stores in the liver and from the recycling of red blood cells in the spleen. There was, however, no significant variation in △M observed for the urine during the same period. This inconsistency between the values of △M for the kidney and the urine may have resulted from low concentrations of iron metabolites with lower magnetic properties.

The re-emergence of MNP metabolites during the chronic phase is also supported by the data from the TEM and Prussian blue staining analyses. The TEM images showed small numbers of dark particles in the lumen of glomerular blood vessels at 8 h and 1 d following MF injection. Small numbers of the dark particles were also observed in the cytoplasm of renal tubular cells in the TEM images collected 4 wk post-injection, indicating that the levels of MNP metabolites present were likely below the threshold detectable by using Prussian blue staining (Figure 7C). These findings are also supported by the results of a previous study [12].

### Conclusion

By using this proposed novel SSB, the outcome of injected magnetic Fe_3_O_4_ nanoparticles was examined noninvasively. Most of the injected MNPs were transported into the heart and other organs. After reaching the organs that are rich with macrophages, the MNPs were phagocytized and biodegraded. The metabolites originating from the MNPs were transported in the blood from the organs to the kidneys, from which they were excreted in the urine. Increased levels of iron metabolites in the blood corresponded with increased numbers of red blood cells and increased levels of transferrin in the serum. In addition, the MNP metabolites were recovered during hemoglobin recycling in the spleen. Correlations between the *in vivo* and *in vitro* test results indicate the feasibility of conducting SSB examinations for measurement MNP and erythrocyte concentrations, implying future clinical applications of SSB.
